# Supplementary material for: Transcriptional profiles of antidepressant resistance across the corticolimbic pathway of chronically stressed mice
Source: Neuropsychopharmacology. 2026 Feb 23;51(7):1279–89. doi: 10.1038/s41386-026-02366-6 (PMC13212567; doi:10.1038/s41386-026-02366-6)
Supplement: Supplementary file 1 — SUPPLEMENTAL MATERIAL [file 41386_2026_2366_MOESM1_ESM.docx]

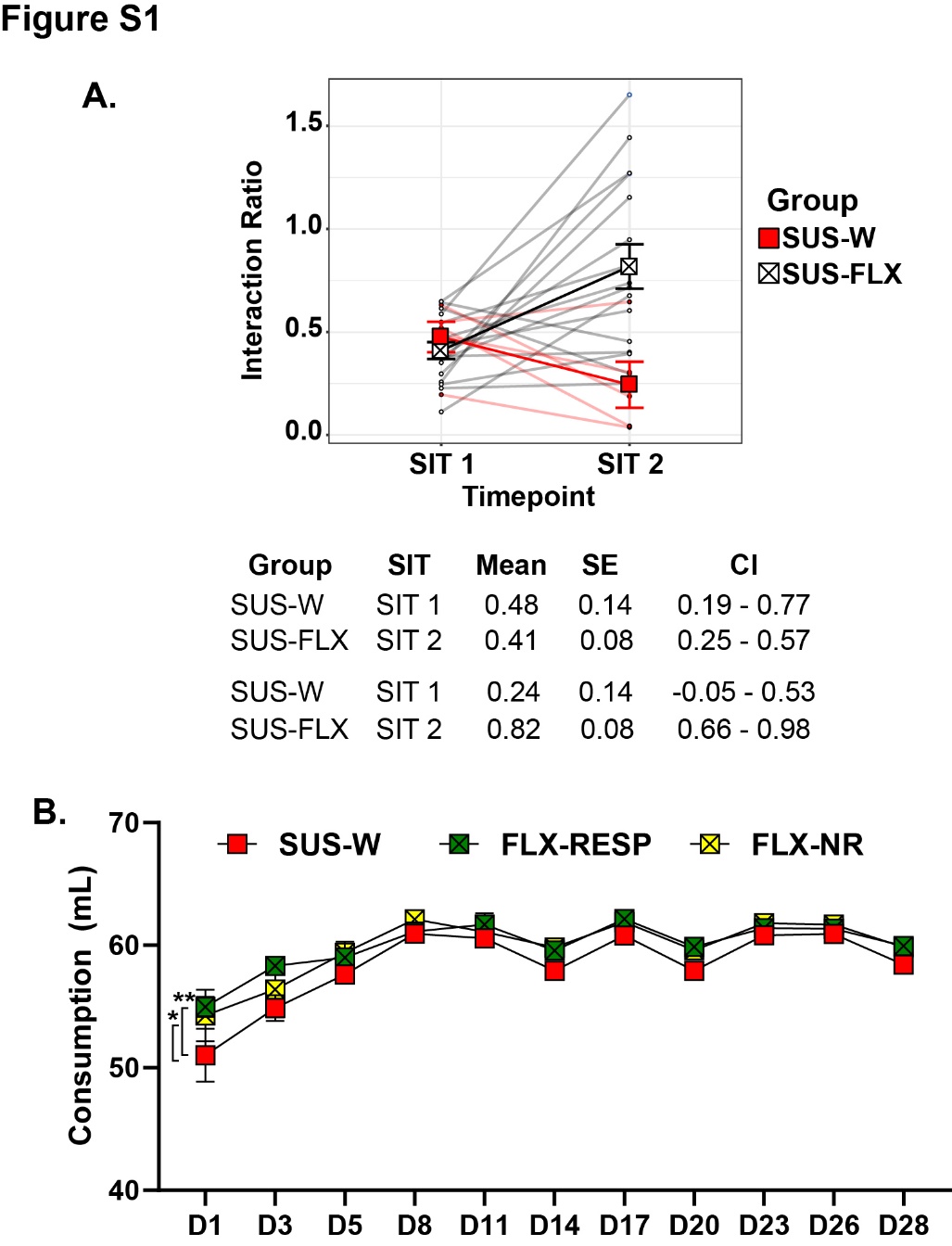


Figure S1. FLX treatment in SUS mice to CSDS. Related to Figure 1. (A) Social interaction ratio across SUS mice treated with water (SUS-W) or with FLX in the drinking water. (B) Two-days average water consumption across the 28 days of FLX treatment. Two-way ANOVA: Group: F(2,264)= 11.20; p<0.0001; Day: F(10,264)= 24.88; p<0.0001; Group by Day interaction: F(20,264)= 0.5242; p=0.95. Tukey’s test: Increased water consumption in FLX-RESP (*p<0.01) and FLX-NR (*p<0.05) on Day 1.


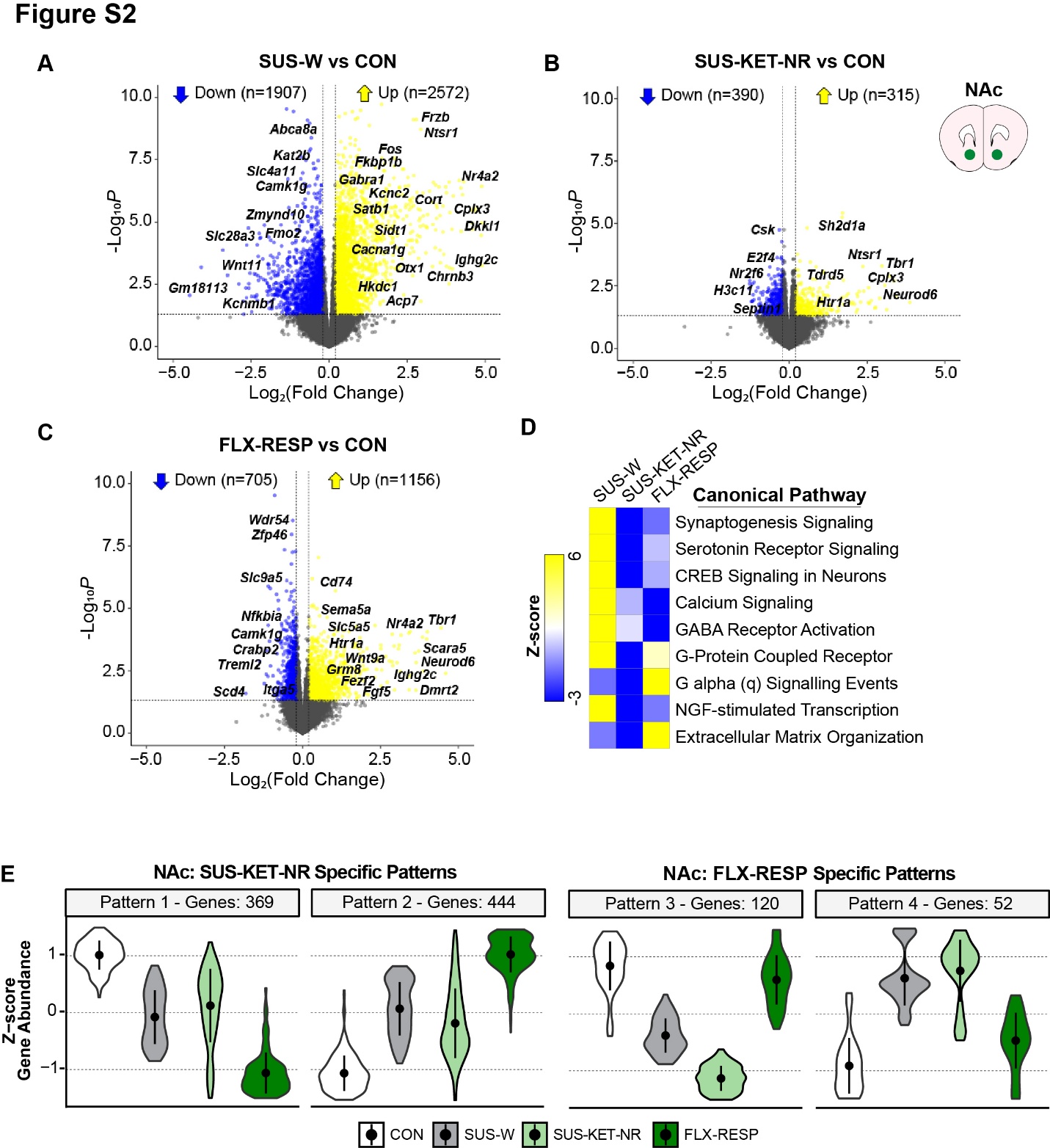


Figure S2. Transcriptional signatures of single antidepressant treatments in NAc. Related to Figure 2. (A) Volcano plot showing differentially-expressed genes (DEGs) in SUS-W compared to CON. (B) Volcano plot showing DEGs in SUS-KET-NR versus CON. (C) Volcano plot showing DEGs in FLX-RESP versus CON. DEG criteria: Log2(fold change) > |0.20|, nominal p-value of <0.05, and false discovery rate <0.1. Blue and yellow represent downregulation and upregulation, respectively. (D) Ingenuity Pathway Analysis (IPA) showing top canonical pathways for each group ranked by Z-score. (E) Violin plots show z-scored gene abundance for SUS-KET-NR specific clusters (left) or FLX-RESP (right). These clusters include genes uniquely regulated in SUS-KET-NR or FLX-RESP mice.


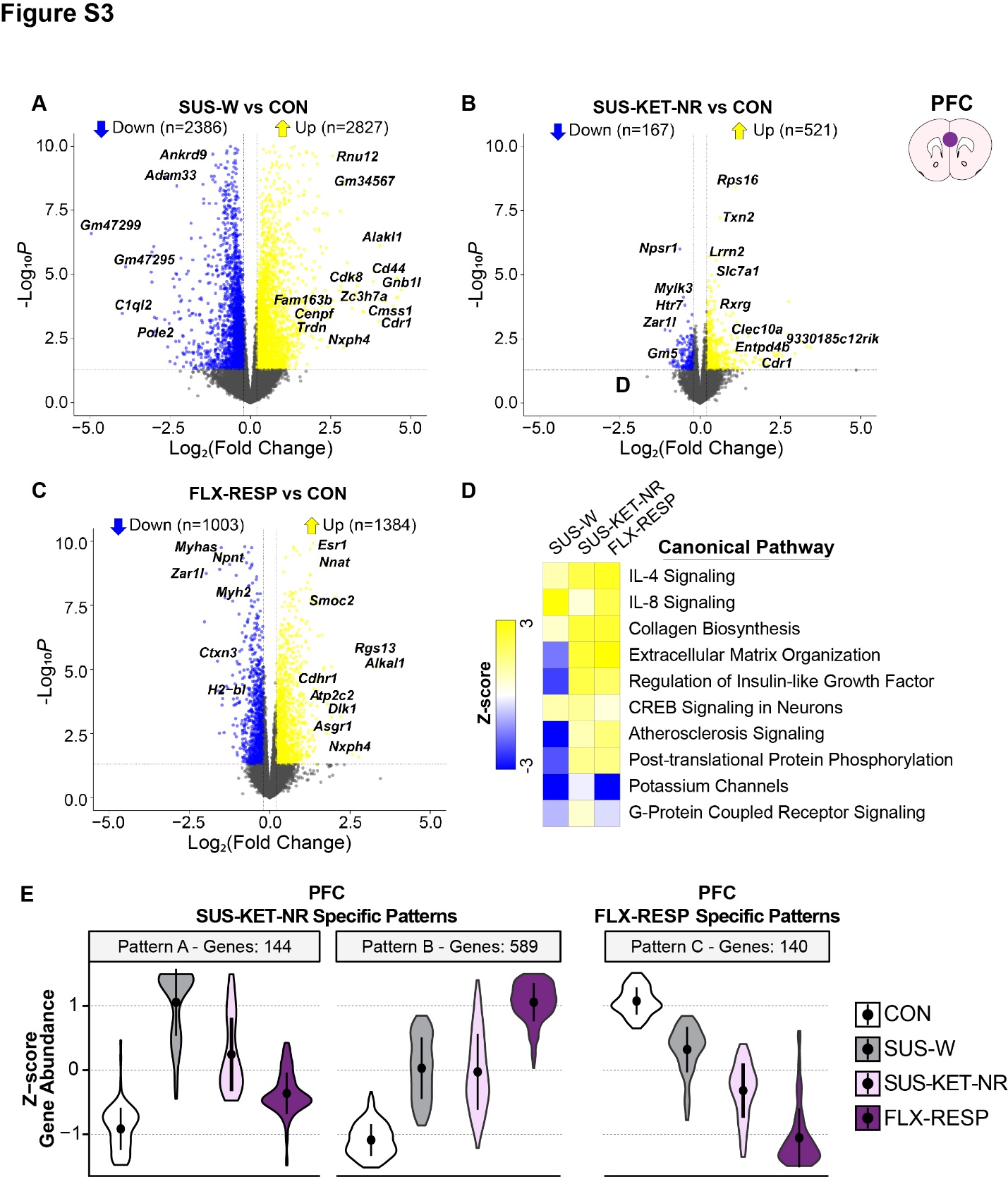


Figure S3. Transcriptional signatures of single antidepressant treatments in PFC. Related to Figure 2. (A) Volcano plot showing differentially-expressed genes (DEGs) in SUS-W compared to CON. (B) Volcano plot showing DEGs in SUS-KET-NR versus CON. (C) Volcano plot showing DEGs in FLX-RESP versus CON. DEG criteria: Log2(fold change) > |0.20|, nominal p-value of <0.05, and false discovery rate <0.1. Blue and yellow represent downregulation and upregulation, respectively. (D) Ingenuity Pathway Analysis (IPA) showing top canonical pathways for each group ranked by Z-score. (E) Violin plots show z-scored gene abundance for SUS-KET-NR specific clusters (left) or FLX-RESP (right). These clusters include genes uniquely regulated in SUS-KET-NR or FLX-RESP mice.


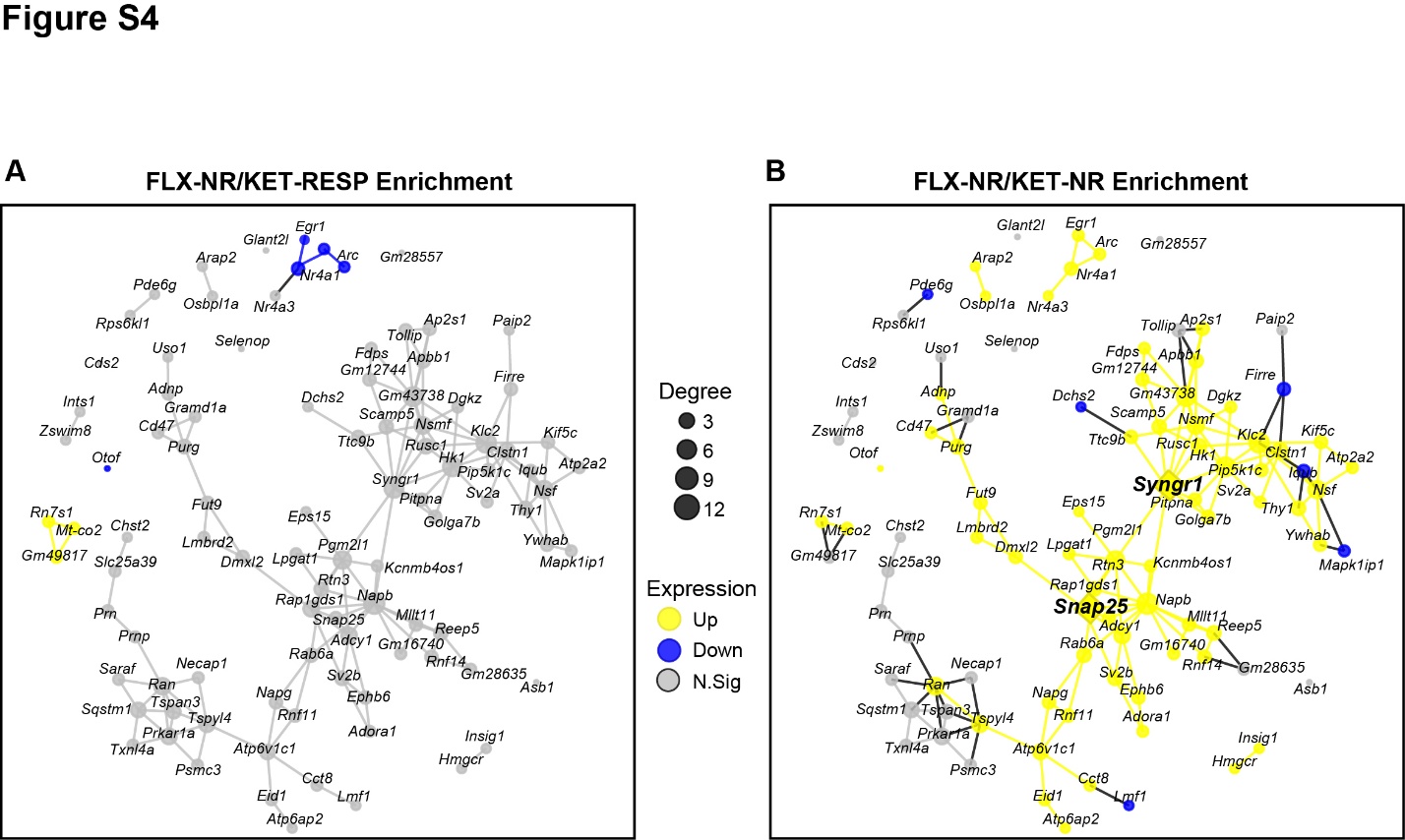


Figure S4. Visualization of gene networks that illustrate key hub genes in the NAc associated with antidepressant resistance. Related to Figure 5. (A) Network structure of cluster 10 showing defined hubs as diamonds and non-hub nodes as circles, both sized by degree of connections. Differentially expressed genes (>20% FC, p<0.05) from the SUS-W condition are overlaid onto FLX-NR/KET-RESP, and (B) FLX-NR/KET-NR group networks (upregulated, yellow shading; no change, gray shading), demonstrating near complete activation of this network.

Table S1.

**IPA output.** Contains the canonical pathways and upstream regulators for each differential expression comparison using the Ingenuity Pathway Analysis.

Table S2.

**Pattern data.** Contains gene assignment for each identified pattern along with Z-scored expression data.

Table S3.

**MEGENA output.** Contains gene to gene correlation weights and the module assignment for each gene following MEGENA analysis of all experimental groups.
